# Successful management of severe allergic reactions to platelet transfusion with omalizumab: A case report

**DOI:** 10.1097/MD.0000000000027724

**Published:** 2021-11-05

**Authors:** Yeonjoo Choi, Ja Min Byun, Inho Kim, Jin Hyun Park, Ki Hwan Kim, Jin-Soo Kim, In Sil Choi, Min-Suk Yang, Hyunkyung Park

**Affiliations:** a Department of Hematology and Oncology, The Catholic University of Korea, St. Mary Incheon Hospital, Incheon, Republic of Korea; b Department of Hematology and Oncology, Seoul National University Hospital, Seoul, Republic of Korea; c Department of Hematology and Oncology, Seoul National University Boramae Medical Center, Seoul, Republic of Korea; d Department of Allergy and Clinical Immunology, Seoul National University Boramae Medical Center, Seoul, Republic of Korea.

**Keywords:** anaphylaxis, hypersensitivity, omalizumab, platelet, transfusion

## Abstract

**Rationale:**

An allergic transfusion reaction is a common side effect of transfusions of red blood cells. Using washed red blood cells is the most effective method for preventing such a reaction. However, the availability of other washed transfusion components, including platelets, is limited.

**Patient concerns:**

A 69-year-old patient with acute myeloid leukemia progressed from myelodysplastic syndrome and was treated with azacitidine. She experienced a minor reaction to platelet transfusion that initially responded to the administration of corticosteroids and antihistamines. However, she worsened even after subsequent preventive treatments and was referred to the emergency department due to anaphylaxis. The patient developed hypotension, chest pain, and dyspnea 10 minutes after the initiation of platelet transfusion.

**Diagnoses:**

She was diagnosed with platelet-induced anaphylaxis.

**Interventions:**

In an attempt to prevent anaphylaxis, 150 mg of omalizumab was prescribed 1 week prior to transfusion. However, she experienced anaphylaxis again and was administered intramuscular epinephrine. For the following transfusion, we treated her with a 300 mg dose of omalizumab 24 hours before the transfusion.

**Outcomes:**

She tolerated well and continued to receive further chemotherapy and platelet transfusion with premedication.

**Lessons:**

This case suggests that omalizumab is a good candidate for the management of severe allergic transfusion reactions.

## 1 Introduction

Allergic transfusion reactions (ATRs) are common side effects of transfusion. Many different blood components can induce such reactions, and they are more common in cases of platelet or plasma transfusion due to the activation of leukocytes and the accumulation of cytokines when these transfused products are stored at room temperature.^[1]^ The incidence of ATRs is 0.3% to 6% for platelet transfusion and 1% to 3% for plasma transfusion.^[2]^

The etiology of ATRs remains unclear. However, both the allergic characteristics of the patient receiving the transfusion and the cellular and plasma components of the transfusion products can contribute to the development of ATRs,^[3]^ specifically type 1 hypersensitivity reactions in which immunoglobulin E (IgE) interacts with components in the donor plasma.^[3]^ Manifestations vary from mild allergic reactions to severe anaphylaxis. Specific medical conditions, such as IgA or haptoglobin deficiency, can make patients more susceptible to anaphylaxis.^[4,5]^ Mild reactions can be managed with corticosteroids or antihistamines, but severe anaphylaxis must be treated with prompt injection of epinephrine.^[6]^ To prevent ATR recurrence, pretreatment with antihistamines and systemic corticosteroids can be used in mild cases; further, the use of washed blood components has been shown to be helpful in some cases.^[6]^ However, there are no definitive guidelines to prevent the recurrence of severe ATRs, especially those induced by platelet transfusion.

Omalizumab is a recombinant, humanized, monoclonal anti-IgE antibody that binds to circulating IgE molecules and prevents IgE binding to high- and low-affinity IgE receptors (FcεRI and FcεRII) on effector cells.^[7]^ It is widely used for the treatment of severe asthma and refractory chronic urticaria.^[7]^ In limited cases, omalizumab has been used to manage anaphylaxis.^[8]^ We report a case of platelet-induced anaphylaxis that was successfully managed with omalizumab.

## 2 Case presentation

A previously healthy 69-year-old female patient was diagnosed with myelodysplastic syndrome and refractory anemia with excess blasts-1 in June 2016. Her physical examination revealed no specific findings at the time of diagnosis, but the initial complete blood count was indicative of pancytopenia (white blood cell count, 1040/μL; hemoglobin level, 8 g/dL; and platelet count, 28,000/μL). She received azacitidine chemotherapy (75 mg/m^2^; body weight, 61 kg). After 3 rounds of chemotherapy, the patient achieved complete remission, as confirmed by bone marrow biopsy, and continued the treatment course. However, the pancytopenia worsened despite 32 rounds of azacitidine chemotherapy; a complete blood count on March 13, 2020, revealed a white blood cell count of 590/μL, hemoglobin of 7.1 g/dL, platelet count of 42,000/μL, and absolute neutrophil count of 136/μL. Repeated bone marrow biopsy revealed progression to acute myeloid leukemia with myelodysplasia-related changes.

The patient occasionally had adverse ATRs during transfusion. Initially, mild ATRs occurred after the patient was transfused with red blood cells (RBCs), and they were successfully prevented using washed RBCs. The patient also experienced mild ATRs after platelet transfusion, which were prevented using irradiated leukocyte-depleted platelets and pretreatment with antihistamines (4 mg chlorpheniramine) and systemic corticosteroids (100 mg hydrocortisone) until November 29, 2019. When she was transfused with irradiated leukocyte-depleted platelets on February 11, 2020, she experienced shortness of breath, abdominal cramping, and nausea but no skin manifestations. The patient was referred to the emergency department. Her vital signs were stable without desaturation or hypotension, and her symptoms were relieved after treatment with antihistamines and systemic corticosteroids. Baseline IgA levels were normal at 153.97 mg/dL (normal range: 70–400 mg/dL), serum tryptase levels were normal at 1.4 μg/L (normal range: < 11 μg/L) measured 2 hours after anaphylaxis, and total IgE levels were 62.6 kU/L (normal range: < 100 kU/L).

**Figure 1:**
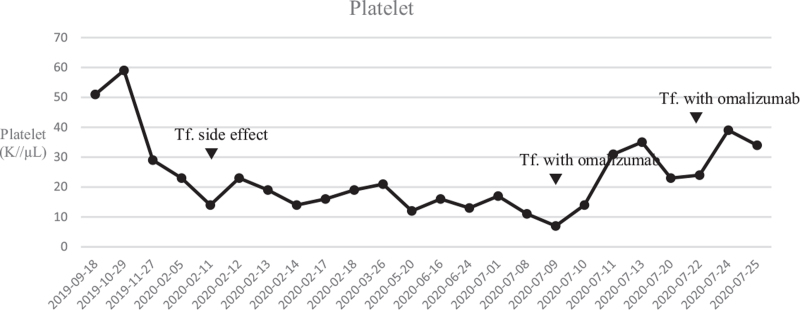
Changes in the patient's platelet counts. PLT = platelet, Tf = transfusion of platelets.

As ATRs were successfully prevented using antihistamines and systemic corticosteroids previously, we planned to increase the dosage and duration of these medications as follows: intravenous administration of 40 mg methylprednisolone at 1, 7, and 13 hours prior to transfusion and 4 mg chlorpheniramine 1 hour prior to transfusion. However, 3 minutes after the initiation of platelet transfusion, the patient complained of chest tightness, and her blood pressure dropped rapidly from 140/90 to 105/75 mm Hg. The patient recovered from shock after intramuscular epinephrine injection. Therefore, we decided to pretreat the patient with omalizumab, an anti-IgE monoclonal antibody, before the next transfusion. Initially, a dose of 150 mg omalizumab was administered to the patient 1 week before the platelet transfusion in addition to systemic corticosteroids and antihistamines administered 13 hours prior to the transfusion. However, chest tightness, abdominal cramps, and shortness of breath with desaturation occurred within 10 minutes after platelet transfusion. For the next transfusion, we administered 300 mg omalizumab 24 hours before transfusion, resulting in the successful completion of the transfusion without recurrent anaphylaxis. Since that time, the patient has tolerated platelet transfusion accompanied by pretreatment with 300 mg omalizumab, systemic corticosteroids, and antihistamines (Fig. 1).

## 3 Discussion

ATRs occur commonly during transfusion. They have been classified into different subtypes, including febrile nonhemolytic reactions, allergic reactions, delayed and hyper-hemolytic reactions, transfusion-related graft-versus-host disease, transfusion-related acute lung injury, and massive transfusion-associated reactions.^[6]^ The management of these different types depends on the specific type of ATR and the blood components transfused. Anaphylaxis is a rare manifestation, with an incidence of < 1%.^[9]^ The use of antihistamines and glucocorticoids is the current gold standard,^[6]^ especially in patients who receive multiple units of platelets (from one or multiple donors), meaning that they are more likely to be exposed to allogenic antibodies and develop reactions easily. Therefore, the removal of plasma components may help prevent ATR. There is some evidence that concentrated apheresis can reduce the incidence of ATRs.^[8,10]^ A washed product is a more effective way to remove plasma under 10%; however, the limited availability of manufacturing is challengeable. ^[8]^We successfully treated our patient with omalizumab, despite the development of anaphylaxis during platelet transfusion in the presence of steroids and antihistamines.

Several mechanisms account for anaphylaxis, one of which is IgA deficiency.^[5]^ According to a previous report by Shi et al,^[8]^ a patient exhibited borderline levels of IgA; however, due to limitations, no additional studies were performed. Our patient had completely normal laboratory results, including initial levels of IgA, tryptase, and other immunoglobulins. The patient initially experienced febrile nonhemolytic transfusion reactions, which were later followed by anaphylaxis. In our case, we did not measure other serum proteins or autoantibodies; therefore, it was difficult to clarify the origin of the ATRs. We hypothesized that our patient produced IgE in response to an unknown plasma protein after receiving transfusion products at certain times. According to one report, multiple components in the plasma, such as complement or haptoglobin, are regarded as candidate triggers of ATRs.^[9]^

Omalizumab is an anti-IgE antibody that binds to free IgE in human plasma or interstitial fluid and reduces sensitivity to allergens.^[11]^ In addition to its antiallergic effects in steroid-refractory asthma, some studies have found that omalizumab can stabilize mast cells and act as an immune-stabilizer in nonallergic diseases.^[11]^ Therefore, omalizumab has been approved for the treatment of chronic urticaria, which is influenced by both allergic and nonallergic mechanisms. It has also been used off-label for the prevention of anaphylaxis during specific allergen immunotherapy, anaphylaxis during mastocytosis, idiopathic anaphylaxis, and transfusion-induced anaphylaxis.^[12]^ The optimal dose of omalizumab for allergic asthma is 75 to 325 mg subcutaneously injected every 2 or 4 weeks; a dose of 300 mg every 4 weeks is commonly used for chronic idiopathic urticaria. However, there is no recommended dose for off-label use. Some case reports of systemic mastocytosis show that multiple doses of 150 or 300 mg omalizumab may effectively manage anaphylaxis.^[13,14]^ In a previous case of platelet-induced anaphylaxis in a patient with IgA deficiency by Shi et al,^[8]^ the patient was pretreated with 150 mg of omalizumab and successfully tolerated platelet transfusion. However, in our case, platelet-induced anaphylaxis was not prevented by pretreatment with 150 mg omalizumab 7 days prior to transfusion. The patient was then treated with 300 mg omalizumab 24 hours before transfusion, which successfully prevented platelet-induced anaphylaxis. The successful prevention of platelet-induced anaphylaxis may be more likely due to the increased dose of omalizumab rather than to the timing of the injection, considering that peak concentrations of omalizumab are reached 7 to 8 days after injection.^[15]^

In conclusion, omalizumab is a promising candidate for the prevention of transfusion-induced anaphylaxis. Further studies are warranted to optimize its dose and efficacy, which will depend on baseline IgE levels as well as the time of drug administration.

## Author contributions

**Conceptualization:** Min-Suk Yang, Hyunkyung Park

**Data curation:** Ja Min Byun, Inho Kim, Hyunkyung Park.

**Investigation:** Jin Hyun Park, Ki Hwan Kim.

**Supervision:** Min-Suk Yang, Hyunkyung Park

**Writing – original draft:** Yeonjoo Choi, Hyunkyung Park, Min-Suk Yang

**Writing – review & editing:** Yeonjoo Choi, Ja Min Byun, Inho Kim, Jin Hyun Park, Ki Hwan Kim, Jin-Soo Kim, In Sil Choi, Min-Suk Yang, Hyunkyung Park.
